# Tomotherapy in synchronous and metachronous bilateral breast cancer: Clinical experience

**DOI:** 10.1002/acm2.14367

**Published:** 2024-04-29

**Authors:** Yernar Orda, Tanzhas Shayakhmetov, Saniya Baiturova, Daulet Berikbol, Rauan Otynshiyev, Aigul Brimova, Bolat Saktashev, Ainur Baisalbayeva, Ainur Samigatova

**Affiliations:** ^1^ Medical Physics Department UMIT International Oncological Center of Tomotherapy: Astana Astana Kazakhstan; ^2^ Radiation Oncology Department UMIT International Oncological Center of Tomotherapy Astana Kazakhstan; ^3^ Clinical Department UMIT International Oncological Center of Tomotherapy Astana Kazakhstan; ^4^ Research Management Department UMIT International Oncological Center of Tomotherapy Astana Kazakhstan

**Keywords:** bilateral breast cancer, metachronous, radiation therapy, synchronous, tomotherap

## Abstract

**Purpose of study:**

The objective of this research is to present our firsthand experience and provide up‐to‐date data for the further study of cases involving simultaneous breast irradiation using helical Tomotherapy, ©Accuray Inc.

**Methods:**

The radical treatment options for bilateral breast cancer are surgery, chemotherapy, and radiation therapy. Being that radiotherapy for bilateral breast cancer is challenging due to limitations in the geometry of modern radiotherapy equipment, helical Tomotherapy was chosen as an appropriate technique of irradiation. The retrospective review focused on the records of patients who underwent bilateral irradiation of the breast or chest wall and regional lymph nodes using helical Tomotherapy.

**Results:**

Only four patients with bilateral breast cancer completed a radiation therapy course in our center from 2018 to 2023. Two patients underwent radical mastectomy with lymph node dissection on both sides before irradiation. For the other two patients, radical mastectomy was done after neoadjuvant chemotherapy. Acute radiation toxicity scoring was based on Common Terminology Criteria for Adverse Events (CTCAE) version 5.0. Only mild adverse effects, such as general weakness and slight skin irritation below Grade 3, were observed, with no instances of skin swelling, dryness, or pigmentation noted. Evaluation of late complications revealed tissue fibrosis in the area of the internal mammary nodes and respiratory failure with various severity. Complications and deterioration in the cardiovascular system were not observed during the follow‐up period, which varied from 3 to 48 months.

**Conclusion:**

Our results show the efficacy of using helical Tomotherapy considering positive outcomes, being that three out of four patients are in remission with low acute toxicity and late complications. There are a small number of articles describing bilateral breast cancer treatment with helical Tomotherapy. On this occasion, our data could contribute to the studies of tolerant doses for organs at risk and improve the parameters of treatment plans for bilateral breast cancer. Since the small sample of patients with bilateral breast cancer limits the study, a larger cohort of patients is essential to obtain statistically reliable results.

## 1 INTRODUCTION

It has been known that synchronous bilateral breast cancer (BBC) occurs in less than 2% of patients,^1^, ^2^, ^3^ but according to recent data, the percentage of cases of breast cancer varies from 1.4% to almost 12%, which is of great research interest.^4^

Radiation therapy in such clinical cases is complicated.^5^ Three‐dimensional conformal radiotherapy (3D‐CRT) is the standard method used for adjuvant irradiation of breast cancer. BBC can be optimally irradiated by using tangential fields when regional lymph nodes are not included in conventional linear accelerators.^6^ However, conventional tangential fields may be insufficient in case of irradiation of regional lymph nodes or if the lumpectomy cavity is located medially.

Therefore, it causes difficulties in reducing doses to organs at risk (OAR) that are located in the beam direction.^6^, ^7^ Multiple electron/photon fields made by conventional linear accelerators are acceptable but raise concerns about multiple field junctions and related dosimetric inhomogeneities.^6^, ^7^ Moreover, in the case of the BBC, each target requires its isocenter. This may result in increases in treatment duration, along with heterogeneous dose distribution around field junctions due to multiple patient setups.

Helical Tomotherapy (HT) combines a continuous rotating linear accelerator with a binary multi‐leaf collimator providing dosimetric superiority as a rotational therapy modality. During radiation, the couch is translated through the gantry while the fan beam is helically delivered and modulated by a binary multi‐leaf collimator. Owing to improved target conformality and OAR sparing compared to conventional 3D or static intensity‐modulated radiation therapy (IMRT) plans, HT allows irradiation in a variety of cases with potential dose escalation.^8^ Integrated megavoltage computed tomography (MVCT) provides 3D images of patient anatomy before irradiation for precise beam delivery and possible adaptive treatment.

In this article, we presented a case series of bilateral breast cancer with the involvement of regional lymph nodes treated using HT.

## 2 CASE PRESENTATIONS

The case series included four BBC patients who received a radiation therapy course for both breasts including lymph nodes using HT. The medical data of the patients were retrospectively analyzed for concomitant diseases, features of the clinical course, as well as acute and late radiation toxicity. Throughout the radiation therapy course, patients were examined once a week, both by a nurse and a radiation oncologist to assess their condition and record acute reactions in case of their occurrence. Patients underwent instrumental examinations (ultrasound, CT) 3 months after radiation therapy to monitor the dynamics of the tumor and the late reactions of normal tissues.

Only four patients with bilateral breast cancer completed a radiation therapy course in our center from 2018 to 2023. Clinical and epidemiological data of patients are presented in Table 1.

**TABLE 1:** Epidemiological data of patients.

Patient	Patient 1	Patient 2	Patient 3	Patient 4
Parameters				
Age	53 years	38 years	29 years	74 years
Heredity	No	No	Maternal parent—breast cancer Paternal parent—stomach cancer	Breast cancer in five generations
Tobacco smoking	No	No	No	No
Alcohol consumption	No	No	No	No
Body‐mass index	35.6 obesity G2	26.8 overweight	24.1 normal	25.9 overweight
Concomitant diagnosis	Hypertension G3, risk 3	–	–	Hypertension G2, risk 3
TNM (right)	T3N0M0	T2N1M0	T1N0M0	T1N0M0
Stage (Right)	IIb	I	IА	0
TNM (left)	T3N0M0	T1bN1aM0	T4N0M0	T1N0M0
Stage (left)	IIb	II	IIIB	0
Synchronus	+	+	–	+
Metachronus	–	–	+	–
NACT	0	3	10	0
ACT	6	6	0	0
AHT	0	0	4	0
HT	Toremifene 4 years	Tamoxifen diphereline	Non‐hormone dependent form	Anastrozole 2 months
IT	0	0	5	0
Surgery	Radical Mastectomy of both sides with lymph dissection	Radical Mastectomy of both sides with lymph dissection	Radical Mastectomy of right side. Subcutaneous mastectomy of left side	Radical Mastectomy of both sides
Histology	Invasive lobular carcinoma on both sides of the 1st degree	Invasive multicentric carcinoma with invasion to skin and nipple tissue	Infiltrative carcinoma with invasion into the muscle tissue of right side. Partially infiltrative carcinoma of left side	Intraductal papilloma with intraductal carcinoma of right side. Encapsulated atypical papillary carcinoma of left side
IHC	ER‐3 g PR‐3 g Her2neu‐neg Ki67 – 3%	ER‐7 g PR‐8 g Her2neu‐neg 1+ Ki67 – 60%	ER‐0 g PR‐0 g Her2neu‐neg Ki67 – 80%	ER– 8 g PR – 8 g
Follow‐up period	48 months	11 months	36 months	15 months
Outcomes	Remission	Died	Remission	Remission

Abbreviations: ACT, adjuvant chemotherapy; AHT, adjuvant hormone therapy; IHC, Immunohistochemistry; IT, immunotherapy; NACT, neoadjuvant chemotherapy.

Two patients underwent radical mastectomy with lymph node dissection on both sides before irradiation. For the other two patients, radical mastectomy was done after neoadjuvant chemotherapy (NACT).

**FIGURE 1:**
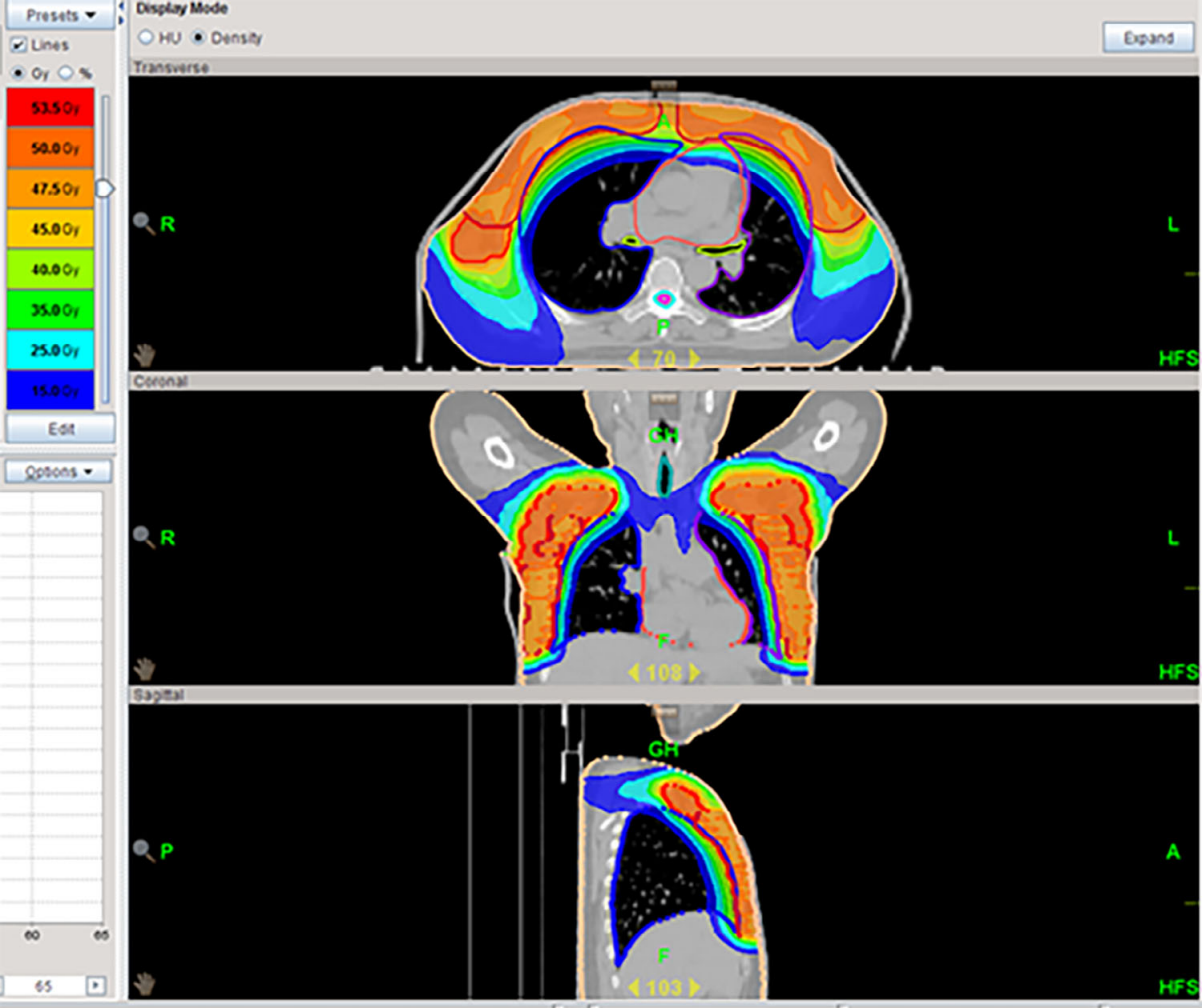
Bilateral breast cancer patient's dose distribution.

Treatment planning was performed with the Tomotherapy planning station, version 5.1.1.6 © Accuray Inc. The main goal of treatment planning is to cover the planned target volume (PTV) with a prescribed dose wherein preserve healthy tissues and organs. Clinical target volume (CTV) involved the postoperative area in the projection of both mammary glands including I, II, and III groups of axillary lymph nodes and internal mammary nodes (IMN). A 5 mm CTV to PTV margin was used for both breast and lymph nodes, excluding 3 mm of skin surface. The upper border was at the level of the upper edge of the clavicle, the lower border was 2 cm from the intrathoracic field, the medial border was the middle of the sternum, and the lateral border was 2 cm mid‐axillary line. A tissue‐equivalent bolus of 5 mm was used to irradiate the chest wall of the patients who underwent a radical mastectomy. The bolus is intended to optimize the coverage of the chest wall by the 95% isodose, thereby increasing the surface radiation dose.^9^ The total dose of 50 Gy was prescribed for each PTV at standard fractionation, that is, 2 Gy per fraction. Due to the inclusion of regional lymph nodes on both sides, patients had considerable radiation area (Figure 1). All plans were calculated using TomoHelical mode. TomoHelical is the original beam delivery method used in the Tomotherapy System and provides rotational delivery of a fan beam.^10^

Treatment plans were calculated using the Tomotherapy planning system and international recommendations (QUANTEC—Quantitative Analysis of Normal Tissue Effects in the Clinic, 2010) were taken into account for OAR doses. The dose limits for lungs were V_5Gy _< 60%, V_20Gy _< 30%, and V_25Gy _< 10%, V_40Gy _< 5% for heart. Regarding PTV coverage conformity and homogeneity indices as well as the V_90%,95%, and 107%_ of the prescribed dose were taken into consideration.

The analysis of specific dosimetric parameters for OAR was performed. Patient‐specific quality assurance was performed using Delta4 Phantom+, Scandidos, Sweden. Dose deviation and distance to agreement criteria for gamma analysis were selected as 3% and 3 mm, respectively. The results of dosimetry data regarding dose limits for OAR as well as PTV coverage and treatment planning settings used for dose optimization are presented in Table 2.

**TABLE 2:** Dosimetric data and plan parameters.

Parameter	Patient 1	Patient 2	Patient 3	Patient 4
Total lung V5(%)	59.22	60.94	47.64	56.13
Total lung V20 (%)	21.44	22.59	25.87	28.19
Total lung mean (Gy)	13.17	13.35	12.77	14.92
Heart V25 (%)	8.21	10.5	10.1	4.68
Heart V40 (%)	0.99	1.24	1.05	1.12
Heart mean (Gy)	8.84	10.19	10.62	13.07
Esophagus mean (Gy)	6.85	5.76	5.13	6.58
Liver mean (Gy)	7.25	4.11	3.24	4.87
PTV_V90_ (%)	99.99	99.97	99.7	99.97
PTV_V95_ (%)	99.92	96.97	98.5	99.5
PTV_V107_ (%)	0	0	0	0
PTV_D2_ (Gy)	50.98	51.58	51.36	51.46
PTV_D98_ (Gy)	48.75	47.09	47.84	48.28
Conformity index	1.27	1.25	1.12	1.21
Homogeneity index	0.04	0.09	0.07	0.06
Beam on time (min)	10.9	9.3	13.7	13
Pitch value	0.43	0.43	0.264	0.264
Modulation factor	2.6	2.7	2.4	2.3
Field size (cm)	2.5	2.5	2.5	2.5
PTV volume (cc)	1421.52	1358.04	1683.1	1882.56

In this study, the conformity index is calculated according to ICRU report 62 and it is the quotient of the treated volume and the planning target volume (TV/PTV).^11^ Treated volume is defined as the tissue volume that receives at least the dose selected and specified by the radiation oncology team as being appropriate to achieve the purpose of the treatment, that is, the 95% isodose.

$$CI=\frac{TV}{PTV}\;$$

PTV, planning target volume; TV, treated volume.

The homogeneity index was calculated by following formula^12^:

$$HI=\frac{D_{2\%}-D_{98\%}}{D_{50\%}}$$

Lower HI values are indicative of a more homogeneous target dose. The near minimum D98% and near maximum doses D2% are doses received by 98% and 2% of the PTV volume.

Acute radiation toxicity scoring was based on Common Terminology Criteria for Adverse Events (CTCAE) version 5.0. Only mild adverse effects, such as general weakness and slight skin irritation below Grade 3, were observed, with no instances of skin swelling, dryness, or pigmentation noted.

Evaluation of late complications revealed tissue fibrosis in the area of the internal mammary nodes and respiratory failure with various severity. However, patients did not have any lung diseases, including emphysema before radiation therapy. Complications and deterioration in the cardiovascular system were not observed during the follow‐up period, which varied from 3 to 48 months. Fully assessing late complications poses a challenge due to the limited duration of observation. Acute and late reactions of patients are presented in Table 3.

**TABLE 3:** Acute and late reactions.

Reaction	Patient 1	Patient 2	Patient 3	Patient 4
Acute				
Skin swelling	0	0	0	0
Skin irritation	1	2	1	1
Skin dryness	0	0	0	0
Skin pigmentation	0	0	0	0
Weakness	2	2	2	1
Late				
Tissue fibrosis	1	0	0	0
Heart complications	0	0	0	0
Secondary malignancies	0	0	0	0
Respiratory insufficiency (pneumonitis)	1	1	2	0

## 3 DISCUSSION

In Kazakhstan, as in other countries, breast cancer takes first place in the structure of malignant neoplasms among women. According to our Ministry of Health data on average, up to 5000 patients with breast cancer are diagnosed every year, and up to 1200 women die. Although BBC is a relatively uncommon type of breast cancer, its radiation therapy with conventional techniques is cumbersome, hence modern techniques and approaches must be applied for better irradiation. This is the first clinical experience of Tomotherapy implementation in the BBC treatment in Kazakhstan.

In this study, we analyzed epidemiological, clinical, and dosimetric data of BBC patients and reported our findings.

The risk of developing oncological diseases may be increased by the presence of concomitant pathologies, genetic predisposition, exposure to environmental radiation, as well as tobacco and alcohol consumption. Data analysis showed patient №4 had genetic mutation BRCA1 (breast cancer gene 1), and from anamnesis vitae breast cancer was reported in five generations by maternal side. Both parents of patient №3 had cancer, but genetic analysis was not performed. None of the patients consumed alcohol or tobacco, two patients were overweight, while one patient had obesity grade 2. Overweight is noted in 75% of cases in our cohort of patients, which goes along with research that has proven excess body weight is a risk factor for breast cancer.^13^ Two patients presented with arterial hypertension, which was effectively managed pharmacologically, ensuring the absence of complications during the course of radiation therapy.

Currently three out of four patients are in remission with primarily different stages and characteristics. All patients who were in remission did not have initial lymph node invasion, which indicates a favorable prognosis of the tumor process. Treatment included standard neoadjuvant and adjuvant chemotherapy regimens with hormone replacement therapy in three cases, one patient received immunotherapy.

Patient №2 did not receive any specific therapy for 1 year after determining diagnosis, throughout the following 2 years she received hormone therapy. During hormone therapy tumor progression was noticed, therefore six courses of NACT were administered. The course of radiation therapy using Tomotherapy was performed subsequently. Tumor relapse with metastasis revealed after 8 months of remission, eventually leading to a fatal outcome.

According to the QUANTEC recommendations on dose limits our treatment planning results demonstrate the following data: average V20Gy in total lungs was 24.52% (range 21−28), the average mean lung dose was 13.55 Gy, the average mean heart dose was 10.7 Gy (range 8.8–13).

Features of the Tomotherapy HD allowed us to achieve a conformal and uniform dose distribution of the PTV while excluding hot points. Considering large PTV volumes and 2.5 cm field width the beam‐on time averaged over 10 min (11.72).

This paper reports our initial experience of BBC treatment using Tomotherapy HD linac. Owing to design features, Tomotherapy makes it possible to irradiate both sides of BBC by a single treatment plan without patient repositions.

There are a small number of articles describing bilateral breast cancer treatment with Tomotherapy.^14^, ^15^ On this occasion, our data could contribute to the studies of tolerant doses for OARs and improve the parameters of treatment plans for the BBC. Based on the literature review the obtained average values of doses to OARs during treatment planning in this study were comparable with the results of Orit Kaidar‐Person et al., moreover for some OAR, specifically for lungs, heart and liver doses were even lower.^16^

## 4 CONCLUSION

Our results show the efficacy of using Tomotherapy considering positive outcomes, being that three out of four patients are in remission with low acute toxicity and late complications. Since the small sample of patients with BCC limits the study, a larger cohort of patients is essential to obtain statistically reliable results.

## AUTHOR CONTRIBUTIONS

Yernar Orda–Principal Investigator, Designed the study, Collected and analyzed data, Provided critical revisions to the manuscript, Gave final approval for publication. Tanzhas Shayakhmetov–Contributed to data analysis, Drafted the manuscript, Provided valuable insights into the analytical aspects of the research. Daulet Berikbol–Oversaw clinical data collection, Made the selection of cases, Played a crucial role in ensuring accurate and relevant clinical data. Rauan Otynshiev–Contributed to case selection, Chose the evaluation criteria, Provided expertise in defining the criteria for evaluating cases. Saniya Baiturova–Conducted a comprehensive literature review, Contributed to the selection of criteria, Brought a strong theoretical foundation to the research. Aigul Brimova–Contributed to forming clinical diagnoses, designed research consent form of patients for research, Ensured ethical considerations in patient interactions. Bolat Saktashev–Contributed to forming clinical diagnoses, designed research consent form of patients for research, Ensured ethical considerations in patient interactions. Ainur Baisalbayeva–Contributed to drafting the manuscript, Acted as an independent evaluator of the paper, Provided critical feedback from an objective standpoint. Samigatova Ainur, Acted as an independent evaluator of the paper.

## CONFLICT OF INTEREST STATEMENT

The authors declare no conflicts of interest.
